# Rapid Onset of B12 Deficiency in the Setting of Worsening Multiple Myeloma: Correlations between B12 Deficiency and Multiple Myeloma

**DOI:** 10.1155/2017/6458676

**Published:** 2017-07-31

**Authors:** Karan Seegobin, Satish Maharaj, Grant Nelson, Jeremy Carlson, Cherisse Baldeo, Rafik Jacob

**Affiliations:** Department of Internal Medicine, University of Florida College of Medicine, Jacksonville, FL 32209, USA

## Abstract

A 67-year-old female with a relapse of multiple myeloma after being in remission for approximately 2 years following autologous stem cell transplant presented with worsening pancytopenia, over a three-month period. There were an increase in her monoclonal spike at 3.13 g/dL on serum protein electrophoresis, low serum B12 levels, and positive intrinsic factor antibodies. Three months before, she had normal B12 levels and a significantly lower monoclonal spike of 1.07 g/dL. She was diagnosed with B12 deficiency with pernicious anaemia in the setting of her worsening myeloma. Multiple myeloma (MM) has been linked with B12 deficiency and pernicious anaemia. Several mechanisms have been described regarding the pathogenesis of B12 deficiency in such patients. Increased tumour activity can further perpetuate the development of B12 deficiency in such patients. With regard to our case, the increase in tumour activity and onset of pernicious anaemia could have contributed to the rapid development of B12 deficiency. In contrast to this, rapid development of B12 deficiency could also signify relapse or worsening of the myeloma as seen in our case. Physicians ought to consider B12 deficiency in patients with worsening pancytopenia and myeloma.

## 1. Background

Multiple myeloma is a clonal malignancy of plasma cells characterized by an overproduction of monoclonal antibodies [1]. The IgG and IgM paraproteinemia of the kappa type as well as IgA myeloma have been linked with pernicious anaemia [1–3]. Generation of specific autoreactive antibodies, anti-intrinsic-factor-like activity of the IgM paraprotein, increased tumour burden, immunomodulatory properties of lenalidomide, and disruption of renal mechanisms of B12 absorption are some of the described mechanisms by which B12 deficiency can occur in these patients [1, 4, 5]. Physicians ought to be aware of the association between B12 deficiency and multiple myeloma as its onset can signify worsening of the disease. Further research is needed into the usefulness of B12 levels as a marker of worsening myeloma. We described a case of myeloma that presented with worsening pancytopenia and was found to have B12 deficiency, pernicious anaemia, and worsening of the disease activity, signified by increase in monoclonal paraprotein levels. As paraprotein levels increased, the B12 levels decreased in our case. Further research is needed to show the usefulness of B12 levels in multiple myeloma with regard to disease activity. We further review the mechanisms of B12 deficiency in these patients and discuss the utility of monitoring vitamin B12 levels in these patients.

## 2. Case Presentation

We report a case of a 67-year-old female with multiple myeloma and hypertension who was previously treated with chemotherapy and autologous stem cell transplant with good response, having no evidence of monoclonal gammopathy on serum immunofixation. The patient subsequently had a relapse of the disease two years after the transplant, with reoccurrence of the IgG lambda monoclonal paraprotein on serum immunofixation. The patient did not wish for another stem cell transplant; she was then managed with lenalidomide and dexamethasone which were stopped when she experienced worsening pancytopenia over a three-month period. Despite stopping the medications, the pancytopenia still progressed. She also complained of intermittent rash on both upper extremities over this time which would resolve on its own. On examination, she was not in respiratory distress, with vital signs within normal limits. She had petechial bruising on bilateral upper extremities, as well as the thorax. Other aspects of the clinical examination were noncontributory.

Her white cell count was 2.5 (4.5–11 × 103/uL); absolute neutrophil count 449; haemoglobin (Hb) 6 (12–16 g/dL); MCV 100 (82–101 fl); platelet 7 (140–440 thou/cu mm); reticulocyte count 0.9% (0.5–1.5%); reticulated haemoglobin 44.7 (28.8–37.7 pg); reticulocyte index 0.8%; vitamin B12 94 (211–946 pg/mL); intrinsic factor antibody 11.9 (0–1.1 AU/mL); creatinine 1.07 (0.5–0.95 mg/dL), and serum protein electrophoresis showed a monoclonal spike 3.13 g/dL in the gamma region corresponding to IgG lambda paraprotein. Three months before, she had a monoclonal spike of 1.07 g/dL in the gamma region. Furthermore, her B12 levels were 631.5 pg/ml and 751 pg/ml, respectively, 3 and 24 months before. These results are further outlined in Table 1.

She was diagnosed with B12 deficiency and pernicious anaemia in the setting of relapsed myeloma and started on B12 replacements, together with other supportive treatments including blood and platelet transfusions. The patient did not wish to pursue further chemotherapy and preferred palliative and hospice care. She was discharged after improvement in her cytopenias (Table 2); however she was lost to follow-up thereafter.

## 3. Discussion

Multiple myeloma (MM) is a clonal malignancy of plasma cells characterized by an overproduction of monoclonal antibodies [1]. Clinically, this entity is characterized by skeletal lesions, anaemia, hypercalcemia, and renal failure [1]. The incidence of MM is 6.1/100,000 people per year and increases to 30.4/100,000 people per year in those older than 65 years [1]. The median age of diagnosis of MM is 71 years in Caucasians and 67 years in African-Americans [1].

Multiple myeloma (MM) has been linked with several autoimmune conditions in the medical literature [1]. Yet, the significance of these associations is not well understood [1]. In this case the patient was being managed for a relapse of multiple myeloma when she presented with worsening pancytopenia and was found to have B12 deficiency, pernicious anaemia with worsening disease activity.

There are several case reports of pernicious anaemia developing in patients with multiple myeloma [6]. Some studies established that the incidence of cobalamin deficiency in patients with IgA multiple myeloma and MGUS is approximately 13.6% [1]. Another estimated that the prevalence of pernicious anaemia in patients with MM ranged from 4.3% to 5.8% in 1962 [4]. The research has advocated screening for vitamin B12 deficiency in this population [6]. From our case, screening for B12 deficiency may have had three benefits in first detecting the onset of B12 deficiency, early diagnosis of pernicious anaemia, and earlier detection of worsening of the disease activity.

Our patient had elevated IgG lambda paraprotein. Pernicious anaemia has been reported in a case of IgG and IgM paraproteinemia of the kappa type as well as IgA multiple myeloma. [3]. There are several reported mechanisms for the development of B12 deficiency in patients with myeloma which include the following:

[a] Myeloma triggers intrinsic immune alterations and promotes generation of specific autoreactive antibodies, as reported in cases of ITP developing after myeloma [1]. We postulate that worsening of myeloma demonstrated by the increase in the monoclonal spike in our case may have been an underlying trigger for autoantibody production and subsequent development of pernicious anaemia.

[b] The M protein could have anti-intrinsic-factor-like activity or may in some other way interfere with the normal vitamin B12 absorptive process [4].

[c] Malignant plasma cells may more rapidly consume the body's store of vitamin B12 and, hence, increase the likelihood that a patient develops vitamin B12 deficiency [4]. Bone marrow-derived MM cells were shown to have increased uptake and accumulation of vitamin B12 in culture [4]. Concluded from this report is that one would expect a higher prevalence of vitamin B12 deficiency among patients who have MM and among patients who have larger myeloma burden [4]. In our case the B12 levels had a downward trend over the 24-month period, and the monoclonal paraprotein levels had an upward trend as seen in Table 1. This further supports the theory above that plasma cells contribute to development of B12 deficiency.

[d] Excess free light chains (FLCs), readily measurable by the serum FLC assay, could disrupt the renal proximal tubule receptors megalin and cubilin where B12 is reabsorbed [5]. The authors went on to suggest that the prevalence of B12 deficiency would be higher in plasma cell dyscrasias patients with higher free light chain burden [5].

[e] Immunomodulatory properties of lenalidomide are linked with the development of autoimmune diseases [7]. In one report, there was a 4.3% absolute risk of autoimmune disorders in MM patients treated with lenalidomide especially if they had prior autologous transplant [7] Though autoimmune haemolytic anaemia, idiopathic thrombocytopenic purpura, Evans syndrome, autoimmune thyroiditis, optic neuritis, cutaneous vasculitis, and polymyositis have been reported, to the best of our knowledge, we have not seen any cases of pernicious anaemia with lenalidomide [1]. It is possible that lenalidomide contributed to the development of our patient's pernicious anaemia; however this association would have to be validated in larger studies.

An interplay of all these mechanisms above could have contributed to the development of B12 deficiency in our patient. Furthermore, the increased tumour activity in our case could have contributed to her rapid development of B12 deficiency over three months. Our patient had normal B12 levels 24 months prior to presentation and developed low B12 levels during the three-month period when she had worsening pancytopenia with elevation in the monoclonal spike in the gamma region from 1.07 to 3.03. The likelihood that she was B12 deficient prior to the worsening of her myeloma was unlikely since < 5% of patients with serum vitamin B12 levels > 300 pg/mL have biochemical evidence of vitamin B12 deficiency [4]. Furthermore, a serum vitamin B12 level < 200 pg/mL has a high specificity for vitamin B12 deficiency [4].

Vitamin B12 deficiency is a known cause of pancytopenia [8]. We suspect this would have contributed to the patient's pancytopenia. However, the relapse of myeloma and history of lenalidomide use could have also contributed to this hematologic finding. Failure of the pancytopenia to recover after stopping the lenalidomide and dexamethasone made this agent as the underlying cause unlikely. As discussed above, lenalidomide is known to cause pancytopenia; however the hematological indices would be expected to improve after discontinuation of the drug [9].

MM remains incurable with an important life-expectancy shortening [10]. Although the incorporation of the novel agents thalidomide, bortezomib, and lenalidomide in the front-line therapy has resulted in significant improvement, many patients do not have a sustained response making the treatment of relapsed/refractory MM a real challenge [10]. The duration of responses is limited and all patients will develop progressive disease [10]. In light of its nature to relapse and progress, awareness about the association of multiple myeloma with B12 deficiency and pernicious anaemia is important as these can mimic a relapse of myeloma or even signify the onset of worsening disease activity.

## 4. Conclusion

B12 deficiency and pernicious anaemia can develop in patients with relapsed multiple myeloma. Furthermore, its onset can signify worsening disease activity. The development of B12 deficiency can be rapid; additionally, serum B12 levels may be of use in monitoring disease activity. The pathophysiology of B12 deficiency in these patients is not well understood and there is a need for further research in this area.

## Figures and Tables

**Table 1:** Displaying the trend of B12 levels, CBC, and paraprotein levels.

Laboratory test	Time of presentation	Three months before	24 months before
B12 levels (211–946 pg/mL)	94	631.5 pg/ml	751 pg/ml
WCC (4.5–11 × 103/uL)	2.5	4.67	4.5
Hb (12–16 g/dL)	6	11.7	11.1
Platelet (140–440 thou/cu mm)	7	142	143
Intrinsic factor antibody (0–1.1 AU/mL)	11.9		
Monoclonal spike (0.7–1.60 g/dL)	3.13	1.07	0.4

**Table 2:** Complete blood count on admission and prior to discharge.

Hematologic parameter	On admission	Prior to discharge
White cell count (4.5–11 × 103/uL)	2.5	4.75
Hemoglobin 12–16 g/dL	6	8
Platelet 140–440 thou/cu mm	7	14
